# Karyotype Variability and Inter-Population Genomic Differences in Freshwater Ostracods (Crustacea) Showing Geographical Parthenogenesis

**DOI:** 10.3390/genes9030150

**Published:** 2018-03-08

**Authors:** Radka Symonová, Iva Vrbová, Dunja K. Lamatsch, Jürgen Paar, Renate Matzke-Karasz, Olivier Schmit, Koen Martens, Stefan Müller

**Affiliations:** 1 Department of Biology, Faculty of Science, University of Hradec Králové, 500 03 Hradec Králové, Czech Republic; 2 Department Biology II—Institute for Anthropology and Human Genetics, Ludwig Maximilians University Munich, 82152 Planegg-Martinsried, Germany; ifukova@umbr.cas.cz (I.V.); juergen.paar@gmail.com (J.P.); S.Mueller@lrz.uni-muenchen.de (S.M.); 3 Research Institute for Limnology, University of Innsbruck, 5310 Mondsee, Austria; dunja.lamatsch@uibk.ac.at; 4 Institute of Plant Molecular Biology, Biology Centre CAS, 370 05 Ceske Budejovice, Czech Republic; 5 Royal Belgian Institute for Natural Sciences, Department of Freshwater Biology, Vautier Street 29, B-1000 Brussels, Belgium; darwinula@gmail.com; 6 Department of Animal and Plant Sciences, University of Sheffield, Western Bank, Sheffield S10 2TN, UK; 7 Department of Earth and Environmental Sciences, Ludwig-Maximilian University, 80333 Munich, Germany; r.matzke@lrz.uni-muenchen.de; 8 Department of Microbiology and Ecology, University of Valencia, 46100 Valencia, Spain; schmitol@gmail.com; 9 Institute of Human Genetics, Munich University Hospital, Ludwig Maximilians University Munich, 80336 Munich, Germany

**Keywords:** freshwater ostracods, asexuality, reproductive modes, geographical parthenogenesis, comparative genomic hybridization, chromosome numbers, karyotype

## Abstract

Transitions from sexual to asexual reproduction are often associated with polyploidy and increased chromosomal plasticity in asexuals. We investigated chromosomes in the freshwater ostracod species *Eucypris virens* (Jurine, 1820), where sexual, asexual and mixed populations can be found. Our initial karyotyping of multiple populations from Europe and North Africa, both sexual and asexual, revealed a striking variability in chromosome numbers. This would suggest that chromosomal changes are likely to be accelerated in asexuals because the constraints of meiosis are removed. Hence, we employed comparative genomic hybridization (CGH) within and among sexual and asexual populations to get insights into *E. virens* genome arrangements. CGH disclosed substantial genomic imbalances among the populations analyzed, and three patterns of genome arrangement between these populations: 1. Only putative ribosomal DNA (rDNA)-bearing regions were conserved in the two populations compared indicating a high sequence divergence between these populations. This pattern is comparable with our findings at the interspecies level of comparison; 2. Chromosomal regions were shared by both populations to a varying extent with a distinct copy number variation in pericentromeric and presumable rDNA-bearing regions. This indicates a different rate of evolution in repetitive sequences; 3. A mosaic pattern of distribution of genomic material that can be explained as non-reciprocal genetic introgression and evidence of a hybrid origin of these individuals. We show an overall increased chromosomal dynamics in *E. virens* that is complementary with available phylogenetic and population genetic data reporting highly differentiated diploid sexual and asexual lineages with a wide variety of genetic backgrounds.

## 1. Introduction

European freshwater ostracods (Crustacea) offer an attractive model system to investigate transitions from sexual to asexual reproduction and the co-existence of reproductive modes [1]. Exploring the ostracod morphospecies *Eucypris virens* (family Cyprididae, subfamily Eucypridinae) in so far unprecedented details revealed that diploid (2n) asexual and triploid (3n) asexual lineages have originated multiple times from sexual lineages in southern Europe and North Africa [2,3]. Triploid asexuals, however, dominate habitats in northern Europe, suggesting that their wider geographic distribution does not rest on asexual reproduction, but on an elevated ploidy level increasing their heterozygosity [2]. Moreover, phylogenetic analysis revealed an astonishing cryptic diversity indicating the existence of a species complex with more than 40 cryptic taxa [3]. However, the chromosomal background of mixed reproduction in *E. virens* remained unknown, largely because of difficulties to obtain suitable metaphase spreads from small-sized ostracods.

Freshwater ostracods are micro-crustaceans (typically 0.5–2.0 mm in length with the soft body enclosed in a bivalved calcified carapace) inhabiting a broad range of water bodies [4]. The uniqueness of ostracods lies in a diversification of their reproductive modes in freshwater taxa [5] and in a high frequency of transition from sexual to asexual reproduction [6,7]. Recent ostracods include fully sexual species with various sex ratios (e.g., families Candonidae, Ilyocyprididea, Notodromadidae and Cytheridae [5,8]). On the other hand, there are ancient asexuals, i.e., obligate parthenogens on a long-term scale (Darwinulidae) with estimated asexuality for about 100 million years [9]. Finally, there are groups showing so-called geographical parthenogenesis, which refers to a geographically distinct distribution of closely related sexual and asexual species or to distinct distributions of sexual and asexual conspecific populations [5,6,10]. Typically, the asexual species or populations are the more widely distributed [2,5]. Geographical parthenogenesis, and recently evolved parthenogenesis, are particularly common in ostracods of the family Cyprididae [1,5].

In animals, there is a clear association between parthenogenesis and polyploidy that does not apply for plants, where polypoids are common but also sexually reproducing [11]. This has resulted in usage of the term geographical polyploidy [12,13] reviewed by [14]. Polyploidy is an important evolutionary trait with diverse positive as well as negative consequences [15]. Potential cases of geographical polyploidy have been recorded in ostracods and other micro-crustaceans at high latitudes [2,16,17,18]. However, Rossi et al., reported a different pattern from Sicily, where all-female populations of *E. virens* sampled were entirely polyploid [19].

Both polypoids and parthenogens (asexual populations or asexual species) favor, or are able to endure, marginal habitats with fewer biotic interactions e.g., high latitudes, high altitudes, deserts, islands, ecologically disturbed/ecotonal environments or glacially affected environments [6,10,14]. This might be ascribed to their superior colonization abilities and/or to their enhanced ecological plasticity conditioned by heterosis and polyploidy [2]. Polyploidy and parthenogenesis can also result in an increased rate at which chromosome rearrangements accumulate [20,21]. In this special issue, Liehr et al., investigated a comparable model system of highly variable chromosome numbers related to asexual reproduction [22]. Namely, chromosome numbers in stick insects (Phasmatodea) reproducing partly asexually range between 21 and 88. On the other hand, there are examples where karyotypes remain conservative despite simultaneous occurrence of polyploidy and parthenogenesis [23,24]. The reasons why chromosomes remain conservative in some groups, but not in others, remain elusive so far [23,25], and the situation in ostracods with mixed reproduction is not known.

Chromosome number variability in *E. virens* and in other ostracods showing geographical parthenogenesis has been repeatedly reported [26,27,28,29,30,31]. However, our study is the first one employing methods of molecular cytogenetics in ostracods and across a large part of the faunal area of a single (morpho)species. The so far most comprehensive studies on *E. virens* by Tetart [26,27] included karyotypes of parthenogenetic females. Some populations possessed only acrocentric chromosomes; in some others, one small metacentric chromosome occurred, and in others up to seven metacentric or submetacentric chromosomes have been identified. This author reported chromosome numbers in parthenogenetic females ranging between 20 and 34 chromosomes. Bianchi-Bullini and Bullini reported twelve chromosomes in diploid somatic cells in an Italian parthenogenetic population of *E. virens* (2n = 12) consisting of two metacentric chromosomes of very different length, one submetacentric, one subtelocentric and eight acrocentric chromosomes [31]. These records indicate increased chromosomal dynamics in *E. virens* potentially ascribed to asexual reproduction.

The genome size in ostracods represents another kind of neglected area of their biology. The Animal Genome Size Database comprises merely 18 entries on ostracod species [32]. Other 29 new records of genome size in ostracods were reported recently [33]. These data include *Cypris pubera* (C value 0.88) and *Heterocypris incongruens* (in a 3n female C value = 1.11, in a 4n female C value = 1.42) investigated here, but not *E. virens*. The genome size varies within the family Cyprididae in the range 0.48–3.13. As for *E. virens*, a substantial variation in relative genome size within and between diploid and triploid populations has been linked to nuclear genotypes and mitochondrial clades [2,34].

By investigating the cytogenetic background of the transition from sexual to asexual reproduction, we complemented previous studies [2,3] on the ostracod morphospecies *E. virens* (Cyprididae, Eucypridinae). The key question we aimed to address was whether chromosomes of asexual populations show higher variability than sexuals [35]. Our initial karyotyping of *E. virens* populations across Europe and Northern Africa yielded highly variable chromosome numbers and morphologies that were difficult to interpret. Hence, to obtain a more detailed insight into chromosomal genome organization in this species, we employed comparative genomic hybridization (CGH). To this end, we performed whole-genomic comparisons among different sexual and asexual populations of *E. virens*. Furthermore, we performed inter-species CGH (*Cypris pubera* vs. *E. virens*, both Cyprididae) as a comparative measure for the relatedness between *Eucypris* populations. These CGH comparisons pinpointed an overall high genomic divergence in *E. virens* regardless of its reproductive mode, and indicated potential mechanisms of genome evolution. This corresponds to the exceptional cryptic diversity within the morphospecies *E. virens* previously reported by [2,3].

## 2. Materials and Methods

We implemented this study in the framework of the Marie Curie Research Training Network SexAsex, where results of phylogenetic, ecological, behavioral and molecular investigation were utilized [2,3,36,37,38]. Importantly, this study benefited from identification of reproductive modes in *E. virens* populations performed by members of the framework, which otherwise represents another extensive workload.

### 2.1. Specimens

We studied the genomic divergence in several populations of *E. virens* (Jurine, 1820). The sexual populations were collected in Greece (Corfu, Skripera, Blackpond), Morocco (Sidi Chiker) and Spain (Villarreal de San Carlos, Extremadura). Parthenogenetic females were collected in Italy (Rivalazzetto near Parma, Emilia Romagna) and Spain (Almodovar, Castilla La Mancha, and a locality Triops Pond in Ares del Maestre near Valencia). The Almodovar population occurs about 50 km from a mixed population (comprising both sexually and asexually reproducing females). We also used as out-groups for genome comparisons other species: *C. pubera* O. F. Müller, 1776 family Cyprididae, subfamily Cypridinae, *Heterocypris incongruens* (Ramdohr, 1808) family Cyprididae, subfamily Cyprinotinae, both reproducing with geographical parthenogenesis in Europe [8] and *Bradleystrandesia fuscata* (Jurine, 1820) family Cyprididae, subfamily Cypricercinae with in Europe so far unknown males [8,39]. We collected these species in an oxbow of the river Würm close to Gauting (Bavaria, Germany) and in a rock pool in the Zingaro Mountains (Sicily). Finally, we analyzed one fully sexual species *Cyprois marginata* (Straus, 1821) (family Notodromadidae, subfamily Cyproidinae [8]) collected also in the oxbow of the river Würm. Table 1 summarizes the localities and their GPS coordinates of all species used in this study.

### 2.2. Chromosome Preparations

Specimens treated with colchicine (10 µg/mL in distilled water for 24 h) were individually fixed in a freshly prepared fixative (acetic acid/methanol, 1:1, *v*/*v*). The calcified carapaces were removed under a binocular microscope. Soft body parts were chopped in fixative by a syringe and incubated at room temperature (RT) for 30 min followed by sonication for 10 s (in the water bath Sonorex RK100 (Bandelin, Berlin, Germany) at 35 kHz). The cell suspension was dropped onto a cleaned slide in a humid chamber at 55 °C until the liquid evaporated, and then air-dried at RT overnight. Preparations were stained with 4′,6-diamidino-2-phenylindole (DAPI, Sigma-Aldrich (St. Louis, MO, USA) 70 µg/mL in 4 × Saline-Sodium Citrate (SSC)/0.2% Tween20^®^) at 37 °C for 15 min and mounted in Vectashield (Vector Labs. Inc., Burlingame, CA, USA). After an inspection under a microscope, the slides were stored at +4 °C until further use.

### 2.3. DNA Extraction and Whole Genomic Probe Preparation

We extracted genomic DNA by DNeasy Blood and Tissue kit following the manufacturer’s instructions (Qiagen, Hilden, Germany). DNA was eluted with 400 µL of the Buffer AE. Samples were concentrated at the Vacuum Concentrator (BaVaCo-M, Mini-30, Bachofer, Reutlingen, Germany) to approximately 80 µL. Then, 1 µL of the DNA solution was used for a whole genome amplification (WGA) by means of GenomiPhi kit (GE Healthcare, Buckinghamshire, UK). For CGH experiments, the amplification products were labelled by nick-translation with biotin-dUTP (Roche Diagnostics GmbH, Mannheim, Germany), digoxigenin-dUTP (Roche Diagnostics GmbH) or dinitrophenol-dUTP (DNP; NEN Life Sciences, Boston, MA, USA). The probes were stored at −20 °C for further use.

### 2.4. Comparative Genomic Hybridization 

Prior to the hybridization, the DAPI and Vectashield were removed from the slides by two washes in 4 × SSC/0.2% Tween20 for 30 min each at RT. The chromosome preparations were denatured in 70% formamide/2 × SSC at 72 °C for 90 s, immediately dehydrated in an ethanol series (ice cold 70% and 90%, and RT 100% ethanol) 3 min each and air-dried. Various combinations of labelled genomic DNA probes (1 µg of each labeled WGA product) were ethanol precipitated together with 50 µg salmon sperm DNA (Sigma-Aldrich, St. Louis, MO, USA) and re-suspended in 15 µL of hybridization buffer (50% formamide, 1 × SSC, 10% dextrane sulphate) per fluorescence *in situ* hybridization (FISH) experiment. The probes were denatured at 70 °C for 7 min and pre-annealed at 37 °C for 3 h. Then, 15 µL of each probe mixture was mounted on the slide, covered with 20 × 20 mm cover slip and sealed by Fixogum (Marabuwerke, Tamm, Germany). The hybridization was carried out in a humid chamber at 37 °C for 48 h followed by a stringent wash in 0.1 × SSC for 3 × 5 min at 62 °C. Then, biotinylated probes were detected with fluorochromes avidin-Alexa 488 (Molecular Probes Inc., Eugene, OR, USA) or avidin-Cy5 (Dianova, Hamburg, Germany). DNP-labelled probes were visualized by sequential detection with rabbit anti-DNP and goat anti-rabbit-Alexa488 (Sigma-Aldrich), or goat anti-rabbit-Alexa514 (Molecular Probes) antibodies. Digoxigenin-labeled probes were detected with mouse anti-digoxigenin-Cy5 (Dianova). The hybridized slides were counterstained with DAPI (70 µg/mL in 4 × SSC/0.2% Tween20) in PBS for 15 min and mounted in Vectashield.

We employed a series of measurements before starting with the proper CGH experiments to ensure the correct performance of all hybridizations and their interpretation. Firstly, all fluorochromes used in CGH were tested and validated in previous repeated FISH experiments with the reverse fluorochromes usage. Secondly, we performed the self-self hybridizations in *E. virens* as in other ostracod species to verify signal intensity and quality. Thirdly, we performed several sets of CGH experiments on the gradient of genome divergences (same individual, same population, different populations, different species compared) to check the signal quality on more and less similar genomes. Finally, we performed all reciprocal experiments and their replicates under the same stringency conditions, and two different color schemes in the reciprocal hybridizations to exclude any influence of fluorochromes.

### 2.5. Fluorescence In Situ Hybridization with 28S rDNA

To detect the 28S ribosomal DNA (rDNA) on ostracods’ chromosomes, we first PCR amplified an arthropod 28S rDNA probe using the following primer set purchased with Biotech AG (ee: 5′-ATC CGA CTA AGG AGT GTG TAA CAA CTC ACC-3′ and mm: 5′-GAG CCA ATC CTT ATC CCG AAG TTA CGG ATC-3′). The thermal profile of the PCR reaction was: denaturation 3 min at 94 °C, followed by 35 cycles of: 30 s at 94 °C, 30 s at 53 °C, 1.2 min at 72 °C, and finalized by extension for 10 min at 72 °C (details in [40]).

### 2.6. Microscopy

Chromosome, FISH and CGH images were captured with a cooled charge coupled device (CCD) camera (Photometrics C250/A equipped with a KAF1400 chip (Kodak, Rochester NY, USA)) coupled to a Zeiss Axiophot microscope (Zeiss, Oberkochen, Germany), using a conventional filter set by Chroma Technology, Filters combination 8004 (FITC, TRITC, CY 5 and DAPI) and SmartCapture 2 software (Digital Scientific, Cambridge, UK). We analyzed the black and white and color images with the aid of Adobe Photoshop Version CS2 (Adobe, San Jose, CA, USA).

## 3. Results

### 3.1. Chromosome Numbers and Karyotypes in Different E. virens Populations and Other Species

**Figure 1:**
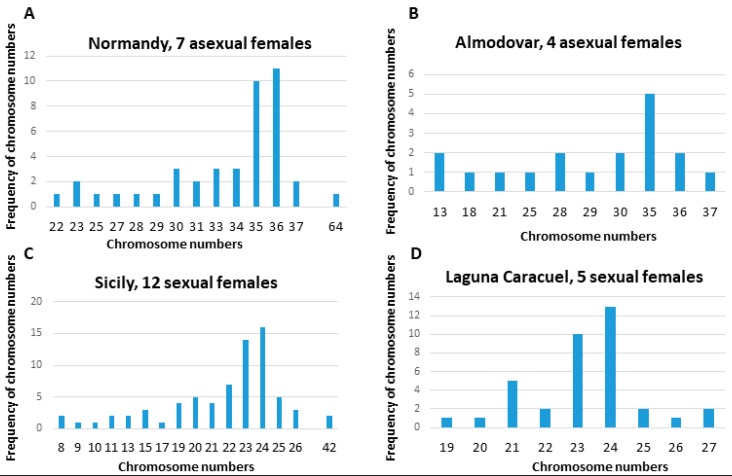
Variation in chromosome numbers in populations of *Eucypris virens*. (**A**) asexual 3n females from France, *n* = 7; (**B**) asexual 3n females from Spain, *n* = 4; (**C**) sexual 2n female from Italy, *n* = 12; (**D**) sexual 2n females from Spain, *n* = 5.

**Figure 2:**
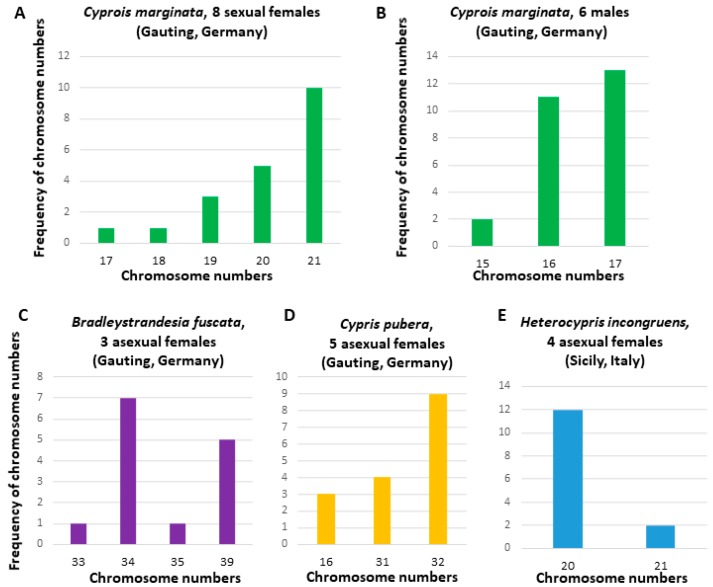
Chromosome numbers in ostracod species of the family Notodromadidae, representing sexually reproducing ostracods, and of the family Cyprinidae, representing asexual and geographically parthenogenetic ostracods. (**A**,**B**) a fully sexual species *Cyprois marginata*, (**A**) females, *n* = 8 and (**B**) males, *n* = 6; (**C**–**E**)/asexual species: (**C**) *Bradleystrandesia fuscata*, asexual females (males are unknown in Europe), *n* = 3; (**D**) *Cypris pubera*, asexual females (geographical parthenogenesis with males confined to Turkey), *n* = 5; (**E**) *Heterocypris incongruens*, asexual females (geographical parthenogenesis with males confined to the east of Europe), *n* = 4.

**Figure 3:**
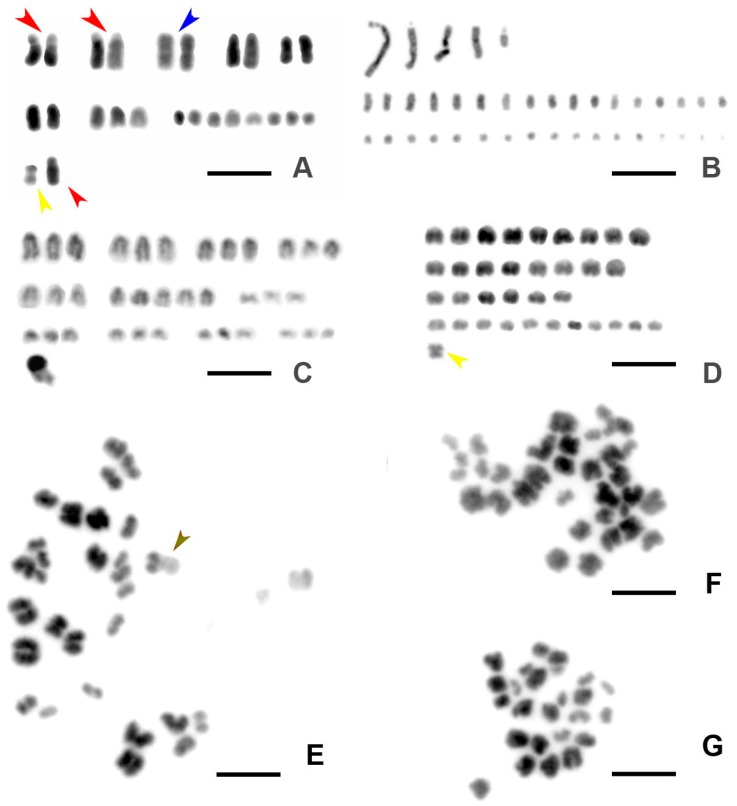
Karyotypes and metaphases of selected populations of *E. virens* analyzed in this study and representing the most important traits of chromosome morphology. (**A**) Sicily, a sexual female, red arrowheads show chromosomes with satellites, blue arrowhead shows a pair of metacentric chromosomes, yellow arrowhead shows a small metacentric chromosome; (**B**) *E. virens*, Sicily, the same sexual female as in (**A**); (**C**) *E. virens*, Valencia, Triops Pond, Spain, an asexual triploid female; (**D**) *E. virens*, Normandy, France, an asexual triploid female, yellow arrowhead shows a small disparate metacentric chromosome; (**E**) *E. virens*, Spain, San Carlos (Bull Pond), a female of a mixed population, brown arrowhead shows a morphologically distinguishable chromosome; (**F**) *E. virens*, France, Normandy, an asexual triploid female; (**G**) *E. virens*, Sicily, a diploid sexual female. Chromosome preparations were stained with 4’,6-diamidino-2-phenylindole (DAPI) and inverted. Scale bars indicate 3 µm.

Our karyotyping in *E. virens* indicated diploid chromosome numbers of approximately 2n = 24, and triploid chromosome numbers of approximately 3n = 36 chromosomes (Figure 1). This was compared with other sexual and asexual ostracod species (Figure 2, details below). However, these counts fluctuated (Figure 3A–F) in asexual (here demonstrated on populations from Valencia and Almodovar (Spain), and Normandy (France) (Figure 1 and Figure 3C,D), as well as in sexual populations (Laguna Caracuel, Spain, and from Sicily; Figure 1 and Figure 3A,B). In the Sicily population, a variability in chromosome numbers and their morphology was recorded. Among cells with chromosome counts mostly 23–24 (Figure 3A), cells with approximately 42 chromosomes (e.g., Figure 3B) have been also identified. Only some chromosomes were morphologically distinguishable among individuals of other populations. The most striking differences were an occurrence of putative satellites (Figure 3A, red arrowheads), a pair of large metacentric chromosomes (Figure 3A, blue arrowhead) and a small disparate submetacentric to metacentric chromosome (Figure 3A, yellow arrowhead). A similar small disparate metacentric chromosome occurred as the only morphologically identifiable chromosome in the triploid population from Normandy (Figure 3D, yellow arrowhead). In the population from Villarreal de San Carlos, another disparate chromosome was morphologically identifiable based on distinctly darker telomeric ends in cells with about 31 chromosomes (Figure 3E, brown arrowhead). This chromosome has not been identified in any other *E. virens* population investigated; however, the quality and also quantity of metaphase spreads did not allow an exact comparison due to the mostly indistinguishable chromosomal morphology. A more detailed karyological evaluation was hampered by the small size (1–2 µm) and morphological uniformity of most chromosomes.

**Figure 4:**
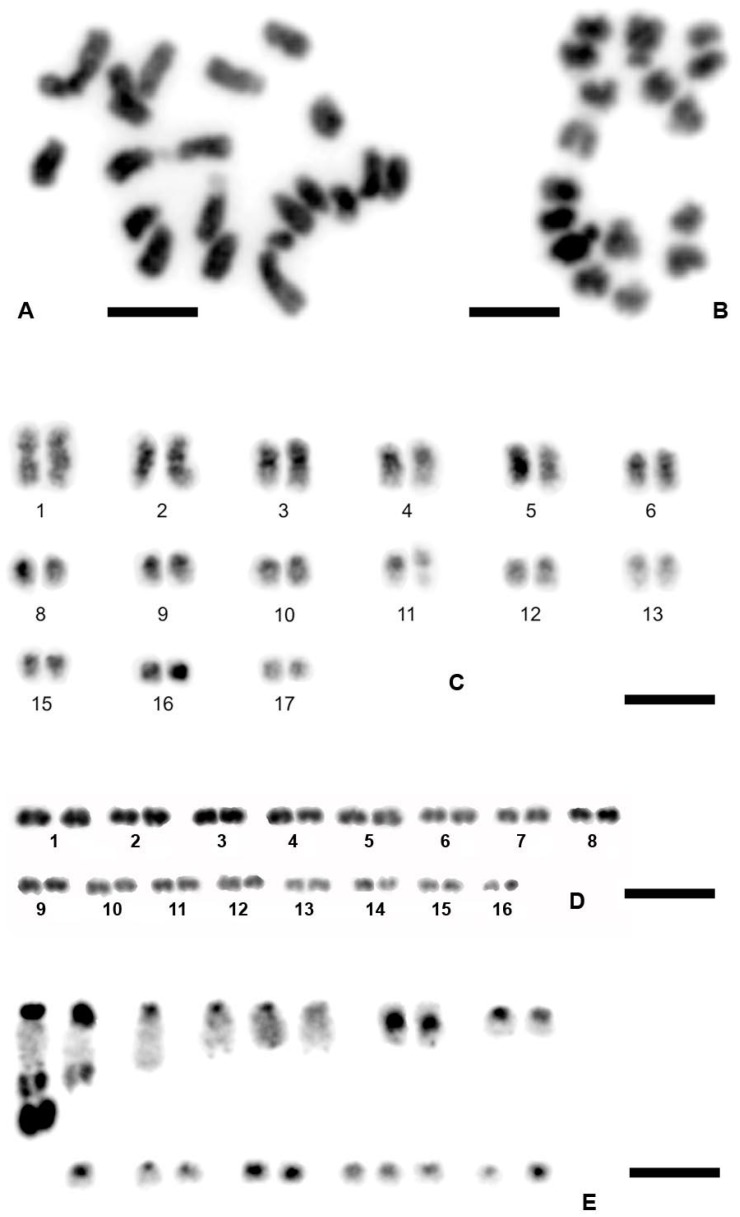
Chromosomes of other non-marine ostracod species. (**A**) *C. marginata*, a sexual female, Gauting, Germany; (**B**) *C. marginata*, a male, Gauting, Germany; (**C**) *B. fuscata*, Gauting, Germany, an asexual female (males are unknown in Europe); (**D**) *C. pubera*, a diploid asexual female, Gauting, Germany; (**E**) *H. incongruens*, Sicily, a diploid asexual female. Chromosome preparations were stained with DAPI and inverted. Scale bars indicate 3 µm.

Furthermore, as a crucial comparative work, we performed an extensive analysis of other non-marine ostracod species representing the same family Cyprididae, where *E. virens* belongs, and the closely related but fully sexual family Notodromadidae. Here, we show examples of results in the fully sexual species *C. marginata* (Notodromadidea) and three asexual species *H. incongruens*, *B. fuscata,* and *C. pubera* (all belong to family Cyprididae). We summarized chromosome numbers of these representative species and their populations in Figure 2. They show narrower ranges in chromosome numbers and mostly left-skewed distributions in chromosomes (Figure 2A,B,D) typically corresponding to chromosome losses during chromosome preparations. Examples of metaphases and chromosome morphology in these species are shown in Figure 4. This part was particularly important to test and to validate the procedure of chromosome preparation applied in *E. virens*, where the obtained results were not satisfactory, in other freshwater, i.e., comparable, ostracod species.

### 3.2. Molecular Cytogenetic Studies of E. virens Genomes by Inter-Population CGH

To better understand this variability in chromosome numbers and their morphology, we performed whole genome comparisons between sexual and asexual females from different populations using CGH. In this step, we could investigate only those *E. virens* populations that yielded chromosome preparations of sufficient quality and cell quantity suitable for CGH experiments. This was the largest limitation of the experimental design enabling to analyze only 2–3 individuals per population and perform only a single replicate with reversed fluorochromes:

#### 3.2.1. Comparative Genomic Hybridization of Almodovar and Rivalazzetto Populations

**Figure 5:**
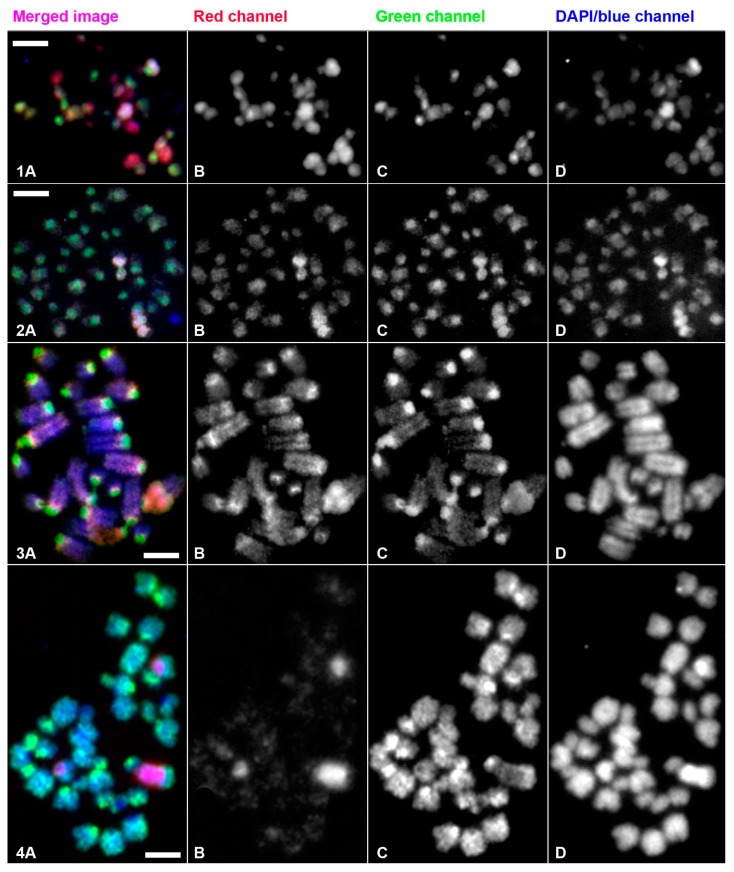
Results of inter- and intra-species comparative genomic hybridization (CGH) experiments: (**1**) chromosomes Almodovar: red Almodovar, green Rivalazzetto; (**2**) reciprocal experiment to the previous one. Chromosomes of *E. virens* Rivalazzetto (Italy), asexual female: red Almodovar (Spain), asexual female, green Rivalazzetto. Note a contamination with microorganisms, presumably microsporidia, in the right corner of the DAPI/blue channel (**2A** and **2D**); (**3**) chromosomes of *E. virens* Valencia (Spain), asexual female: red Corfu (Greece), sexual female, green Sidi Chiker (Morocco), sexual female. Note the higher red signal intensity in three putative rDNA bearing chromosomes in (**3A**–**3B**) and the higher green signal intensity in chromosome centromeres in (**3A** and **3C**); (**4**) chromosomes *B. fuscata,* Gauting (Germany) asexual female (**A**): red *E. virens* (Spain, Bull Pond, sexual female, (**B**), green DNA of *B. fuscata*, (**C**) DAPI-counterstained chromosomes of *B. fuscata*; Scale bars indicate 3 µm.

Genome comparisons of two asexual populations, Rivalazzetto (3n, Italy, DNA labelled in green) and Almodovar (3n, Spain, DNA in red) brought the most interesting results. When whole-genomic DNA probes of both these populations were hybridized to the Rivalazzetto chromosomes (Figure 5(2)A–C), the strongest signals were localized on three chromosomes. DNA probes of both genomes bound evenly to these chromosomes; therefore, they appeared yellow on the merged image (Figure 5(1)A). On the other chromosomes, the Rivalazzetto (green) signal distinctly prevailed over the Almodovar genome; they therefore appeared greenish.

The reciprocal experiment with chromosomes of the Almodovar population showed a completely different pattern (Figure 5(1)A–C). The Almodovar DNA probe (red) bound to all chromosomes; however, four entire chromosomes and some regions of three to four other chromosomes were distinctly highlighted by the red probe compared to the rest of the genome (Figure 5(1)B). The Rivalazzetto DNA probe (green) bound to three to four chromosomes (bright green on the merged image, Figure 5(1)A) more strongly than the Almodovar DNA. This indicates a substantially higher copy number of certain DNA sequence elements on these chromosomes in the Rivalazzetto genome than in the Almodovar genome. Moreover, in seven chromosomes, the Rivalazzetto signal was so suppressed by the overrepresented signal of the Almodovar genome that they appeared invisible in the green channel visualizing the Rivalazzetto signal (Figure 5(1)B red arrowheads).

#### 3.2.2. Comparative Genomic Hybridization of Greece and Morocco Populations

In the next set of experiments, we compared genome probes of sexual females (Greece, red; and Morocco, green) to chromosomes of an asexual female from Valencia, Spain, since chromosome preparations from the two test populations were not available (Figure 5(3)A–C). Both probes highlighted on the less condensed chromosomes mostly centromeres and putative rDNA regions (details in Section 3.3.), but to a different degree. This reveals differences in the copy number of these repeats between these two sexual populations. The centromeric repeats showed higher copy number in the sexual female from Morocco appearing greenish on the merged image (Figure 5(3)A). In contrast to the centromeric regions, two chromosomes bearing the putative rDNA yielded stronger signals of the DNA probe derived from the Greek female (red) (Figure 5(3)A,B).

#### 3.2.3. Interspecies Comparative Genomic Hybridization Comparison of *Eucypris virens* and *Bradleystrandesia fuscata*

This interspecies CGH experiment between *E. virens* (Bull Pond, San Carlos, Spain) and *B. fuscata* (Gauting, Germany) provided a comparison with a more distant genome but still within family Cyprididae. In CGH to *B. fuscata* chromosomes (Figure 5(4)A–C) among predominantly green chromosomes (*B. fuscata* in green and *E. virens* in red), only three chromosomes exhibited a higher proportion of the *E. virens* signal resulting in their overall violet color. The split image (Figure 5(4)B) shows in detail that the *E. virens* DNA bound to a certain fraction of all *B. fuscata* chromosomes.

### 3.3. Fluorescence In Situ Hybridization with 28S rDNA

**Figure 6:**
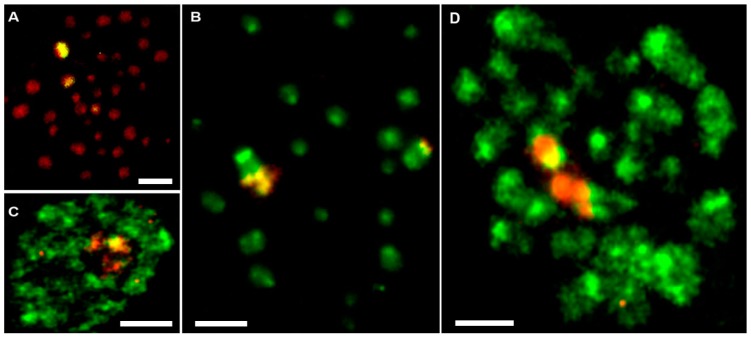
Fluorescence *in situ* hybridization (FISH) with the 28S rDNA probe and interspecies CGH results visualizing rDNA regions. (**A**) 28S rDNA FISH to *E. virens* chromosomes (red) showing three hybridization signals (yellow); (**B**) 28S rDNA FISH to chromosomes of *H. incongruens* (green) with three hybridization signals (yellow); (**C**) interspecies CGH experiments between *E. virens* (red, whole-genome DNA) and *H. incongruens* (green, nucleus and DNA) showing the red signal in the nucleolar region; (**D**) reciprocal CGH experiment to C/on *E. virens* chromosomes (green) with a whole-genome DNA probe of *H. incongruens*. Scale bars indicate 3 µm.

Data involving 28S rDNA are presented in Figure 6. There are results of FISH experiments with the arthropod 28S rDNA probe on *E. virens* (Figure 6A) and on *H. incongruens* (Figure 6B) chromosomes showing three yellow signals in both species. CGH experiments between these two species resulted in a signal (red to orange) in nucleolus on a *H. incongruens* cell nucleus (green) and in three chromosomes (red to orange) in *E. virens* (green), respectively. These experiments are particularly important to verify that the major rDNA fraction is the only one cytogenetically detectable evolutionary most conserved region within the family Cyprididae.

### 3.4. Meiosis in Asexually Reproducing Ostracods

**Figure 7:**
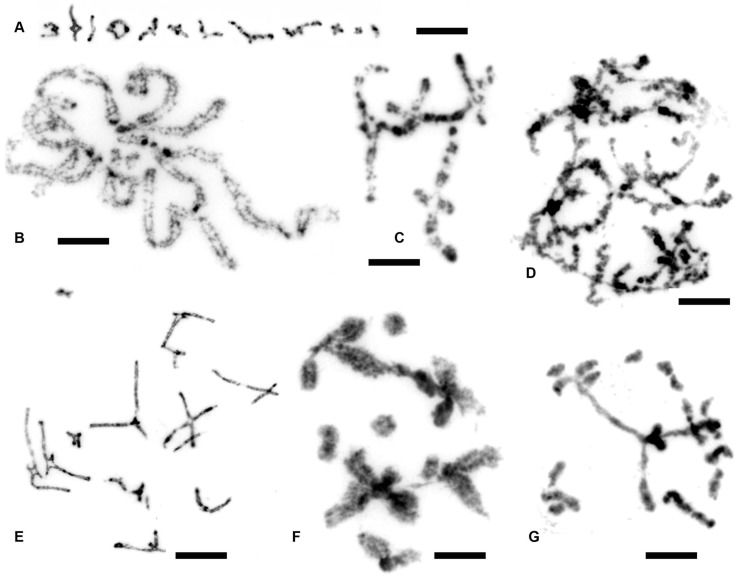
Meiosis in *E. virens* and other asexual species. (**A**) *E. virens*, Spain, Laguna Caracuel, a sexual female showing 12 bivalents; (**B**) *E. virens*, Spain, Almodovar, a probably asexual diploid female; (**C**) *E. virens*, Italy, Sicily, a sexual female, chromosomes chained into a single formation; (**D**) *Bradleystrandesia fuscata*, Germany, Gauting, an asexual female, meiotic chromosomes resembling “lampbrush” chromosomes; (**E**) *E. virens*, Italy, Sicily, a sexual female; (**F**) *E. virens*, Italy, Rivalazzetto, an triploid asexual female; (**G**) *H. incongruens*, Germany, Martinsried, an asexual female. Chromosome preparations were stained with DAPI and inverted. Scale bar indicates 5 µm.

Potential occurrence of meiotic chromosomes in fully asexual species or asexual populations in species with mixed reproduction is an important factor indicating possible mechanisms involved in their evolution. Hence, we focused on meiotic chromosomes particularly in asexual populations of *E. virens* and in fully asexual cypridid species as we show in Figure 7.

## 4. Discussion

To be able to assess the chromosomes numbers variability in *E. virens*, we also analyzed other sexual as well as asexual species (Figure 2). This comparison revealed an overall higher variability in chromosome numbers in *E. virens* and at the same time validated our method of chromosome preparation in ostracods. The outgroup species showed less variable chromosome numbers and less asymmetric distributions of chromosome numbers. Therefore, we can exclude potential radical influences conditioned by the method of chromosome preparation like chromosome losses, etc., although certain artifacts of a smaller extent cannot be ruled out at this stage.

The variability in chromosome numbers reported here for sexual females and asexual females of *E. virens* could not be explained merely by a fundamental numbers (NF) analysis that would indicate potential chromosomal fusions or fissions. This is partly due to the small size of chromosomes and low numbers of metaphases precluding a detailed analysis of chromosomes, and partly due to extreme variability in chromosome morphology among *E. virens* populations irrespective of ploidy and NF numbers. This chromosomal variability cannot be ascribed to chromosome polymorphism as e.g., reported in *Cyclocypris ovum* [41]. In context with the findings reported by [2,3], however, the extreme karyotypic variability may be ascribed to the cryptic diversity of the morphospecies *E. virens*. At the same time, this karyotypic variability within *E. virens* blurs potential differences between sexual and asexual populations, further complicating the already difficult chromosome analysis.

Our previous and here presented fluorescent in situ hybridization (FISH) experiments with 28S rDNA probes combined with CGH indicated that the most conserved regions among cypridid ostracods (*E. virens*, *H. incongruens*, *C. pubera* and *B. fuscata*) are the major rDNA-bearing chromosomes [40]. These usually two to four chromosomes often occur close to each other (cf. Figure 5(2)A,(3)A), which may be a result of satellite association [42]. Therefore, we can consider regions with the most intense fluorescent signals in the present experiments rDNA. Similar observations of large additional regions enriched with rDNA (and telomeric repeats) were reported in parthenogenetic cultures of stick insects [22,43].

The CGH experiment in Figure 5(3)A–C might be indicative of a different rate of rDNA copy number evolution in the populations compared. Namely, a higher copy number of rDNA-bearing regions are apparent in the Greek population (red, Figure 5(3)A,B) in comparison with the Moroccan population. In contrast, in the Moroccan genome (green), the pericentromeric repeats are distinctly over-represented on most chromosomes over pericentromeric repeats of the Greek population (Figure 5(3)A–C). In this CGH experiment, chromosomes belong to an asexual 2n population (Valencia, Spain), and both hybridized genomes originate from geographically remote localities (Greece, red vs. Morocco, green). The inter-species CGH experiments between *E. virens* and *B. fuscata* demonstrate another example of potentially different evolutionary dynamics of repetitive sequences. From Figure 5(4)A–C, it is apparent that these two species share only sequences localized exclusively on three chromosomes in *B. fuscata*. Based on our previous FISH experiments [40] and data presented here (Figure 6), we can assume that these shared sequences represent the 28S rDNA regions and it is possible to recognize the three putative main rDNA-bearing regions with the brightest signal. At this stage, it is premature to say whether only the rDNA is responsible for this FISH pattern or whether any other repetitive element might be interspersed within rDNA. In any case, the signal intensity on the putative main rDNA-bearing chromosomes in *B. fuscata* still appears lower than the signal of *E. virens* DNA. The high signal intensity of *E. virens* on the three chromosomes (Figure 5(4)B) suggests that a higher copy number of these sequences accumulated in the genome of *E. virens*, which is distinctly over-represented signal over the DNA of *B. fuscata* on the merged image (Figure 5(4)A).

Supernumerary B chromosomes represent a possible reason for chromosome number variability. B chromosomes have been reported in sexually (in grasshopper [44,45]) as well as in asexually reproducing insects (in stick insects by [22]). Their presence in parthenogenetic females of ostracods has been mentioned by [26] and later confirmed by the same author [27] reviewed by [46]. B chromosomes are known to consist also of rDNA-bearing DNA among other repeated sequence in invertebrates [47,48]. Therefore, B chromosomes and their polymorphism may contribute to explain chromosome number variation within and among individuals and populations in *E. virens*. Moreover, the morphology of some chromosomes (Sicily and Bull Pond, sexual (2n) and asexual (3n) population, respectively) strongly resembles that of B chromosomes found in grasshopper species *Eyprepocnemis plorans* [49,50]. In this way, the presumable variability in rDNA copy number and number of rDNA-bearing chromosomes and therefore variability of B chromosome numbers might affect the overall chromosome number in a particular population. The non-Mendelian segregation of B chromosomes as well as their origin by translocations from non-homologous chromosomes, mostly autosomes [51], might have also contributed to the chromosome number peculiarities in *Eucypris*. However, a more precise knowledge about the karyotype of *E. virens* will be necessary to confirm the presence of B chromosomes in this species.

The cryptic diversity shown by mitochondrial cytochrome oxidase I (COI) and 16S comparative sequence analysis reported by Bode et al., and its confirmation through corresponding genome size variation [3] provides a plausible explanation of the karyotypic variability (mainly between localities) and diversified patterns of genomic organization in *E. virens*. Moreover, crossing between genomes diverged to such an extent as reported by [3] might also explain the patterns described here. 

## 5. Conclusions

The high chromosomal variability in *E. virens* reported here is in agreement with previously published results [2,3]. The results of karyotyping and CGH experiments also appear to correspond to findings on a substantial variation in genome size within both diploid as well as triploid *E. virens* populations as reported [34]. Namely, the exceptional diversity at the DNA sequence level indicating more than 40 cryptic taxa within this morphospecies may be reflected also at the chromosomal level. We provided initial evidence for an increased cytogenomic dynamics and plasticity in terms of tolerance to aneuploidy and polyploidy probably triggered by asexual reproduction and for a potentially accelerated evolution of repetitive sequences.

Due to the low yield and resolution of ostracod chromosomes, at present, we cannot clearly distinguish between individual variability and population differences. However, we can conclude that the “*E. virens* system” with its mixed reproduction and multiple differentiated lineages shows clearly higher chromosomal dynamics compared to other asexually and sexually reproducing ostracod species [40]. Potential hybridization events between such more and less diverged lineages and unidirectional introgressions might have contributed to and further deepen this condition.

## Figures and Tables

**Table 1:** Overview of population of *Eucypris virens* and other ostracod species analysed.

Species	Country	Population	Sex/Asex	2n/3n	Geographic Coordinates
*E. virens*	Italy	Rivalazzetto (It051)	Asex	3n	N44°49′07″ E10°08′20″
*E. virens*	Italy	Sicily (It064)	Sex	2n	N37°53′27″ E14°22′50″
*E. virens*	Spain	Almodovar	Asex	2n-3n	N38°44′15″ W04°09′11″
*E. virens*	Spain	Valencia, Triops Pond	Asex	2n	N40°25′06″ W00°04′24″
*E. virens*	Spain	Laguna Caracuel	Sex	2n	N38°49’33″ W04°04’01″
*E. virens*	Morocco	Sidi Chiker	Sex	2n	N31°48′42″ W08°32′42″
*E. virens*	Spain	San Carlos, Bull Pond	Mixed	2n-3n	N39°54′22″ W06°03′38″
*E. virens*	Greece	Corfu, Skripera	Sex	2n	N39°41′52″ E19°47′08″
*E. virens*	France	Normandy, Calvados	Asex	3n	N49°11′22″ E00°07′13′′
*C. pubera*	Germany	Gauting, oxbow	Asex	2n	N48°02.455′ E11°22.393′
*H. incongruens*	Germany	Gauting, oxbow	Asex	2n	N48°02.455′ E11°22.393′
*H. incongruens*	Germany	Martinsried, Munich	Asex	2n	N48°06′42.5″ E11°27′47.5″
*H. incongruens*	Italy	Sicily, Zingaro	Asex	2n	N37°53′27″ 14°22′50″
*B. fuscata*	Germany	Gauting, oxbow	Asex	2n	N48°02.455′ E11°22.393′
*C. marginata*	Germany	Gauting, oxbox	Sex	2n	N48°02.455′ E11°22.393′
